# Supplementary material for: Unravelling Vaccine Scepticism in South Tyrol, Italy: A Qualitative Analysis of Personal, Relational, and Structural Factors Influencing Vaccination Decisions
Source: Healthcare (Basel). 2023 Jul 1;11(13):1908. doi: 10.3390/healthcare11131908 (PMC10341176; doi:10.3390/healthcare11131908)
Supplement: Supplementary file 1 [file healthcare-11-01908-s001.zip › healthcare-2409030-supplementary.pdf]

healthcare MDPI

Table S1. Consolidated criteria for reporting qualitative studies (COREQ)

No	*Item*	*Guide questions/description*	*Description/response*
Domain 1: Research team and reflexivity			
Personal Characteristics			
1.	Interviewer/facilitator	Which author/s conducted the interview or focus group?	Koler P, Tauber S, Psaier V.
2.	Credentials	What were the researcher's credentials? *E.g. PhD, MD*	PhD (PK), MSc (ST), MSc (VP).
3.	Occupation	What was their occupation at the time of the study?	Director “Forum Prevention” Psychologist, (PK), Psychologist (ST), Psychologist (VP).
4.	Gender	Was the researcher male or female?	Male (PK), Female (ST, VP).
5.	Experience and training	What experience or training did the researcher have?	Training in motivational interviewing and university background in psychology.
Relationship with participants			
6.	Relationship established	Was a relationship established prior to study commencement?	No.
7.	Participant knowledge of the interviewer	What did the participants know about the researcher? e.g. *personal goals, reasons for doing the research*	They knew it was a commissioned study and were informed about the aims by the information sheet. The researcher had a neutral position on attitudes and this was also communicated.
8.	Interviewer characteristics	What characteristics were reported about the interviewer/facilitator? e.g. *Bias, assumptions, reasons and interests in the research topic*	Profession, impartiality, reasons for the researcher's participation.
Domain 2: study design			
Theoretical framework			
9.	Methodological orientation and Theory	What methodological orientation was stated to underpin the study? *e.g. grounded theory, discourse analysis, ethnography, phenomenology, content analysis*	Grounded theory
Participant selection			
10.	Sampling	How were participants selected? *e.g. purposive, convenience, consecutive, snowball*	Purposive sampling.
11.	Method of approach	How were participants approached? e.g. *face-to-face, telephone, mail, email*	Telephone, email.
12.	Sample size	How many participants were in the study?	Ten.
13.	Non-participation	How many people refused to participate or dropped out? Reasons?	Ten. Reasons: willing to do interview only by phone, feel not to be taken seriously, lack of time, feel not to belong to the target group, ill, later refusal.
Setting			
14.	Setting of data collection	Where was the data collected? e.g. *home, clinic, workplace*	In the offices at the Forum Prävention, at the persons' homes or at places the persons otherwise indicated, such as the youth centre.

15.	Presence of non-participants	Was anyone else present besides the participants and researchers?	No.
16.	Description of sample	What are the important characteristics of the sample? *e.g. demographic data, date*	Seven interview partners were female (70%) and three were male (30%). On average, the persons were 42.5 (range 37-52) years. All interview partners had children. On average, the interviewees had 2.5 children (range = 1-5); they were between 3 months and 21 years old at the time of the interview. Two (20%) interviews were conducted in Italian and eight (80%) in German. Eight (80%) interview partners were assigned to a rural area and two (20%) to an urban area of South Tyrol. Six of the interview participants had a university degree (60%), three attended secondary school (30%), and one completed an apprenticeship (10%).
Data collection			
17.	Interview guide	Were questions, prompts, guides provided by the authors? Was it pilot tested?	There was a question guide that was tested with one person.
18.	Repeat interviews	Were repeat interviews carried out? If yes, how many?	No.
19.	Audio/visual recording	Did the research use audio or visual recording to collect the data?	Audio recording.
20.	Field notes	Were field notes made during and/or after the interview or focus group?	No.
21.	Duration	What was the duration of the interviews or focus group?	Duration of 40 min to 90 min.
22.	Data saturation	Was data saturation discussed?	For an initial assessment in terms of grounded theory: yes.
23.	Transcripts returned	Were transcripts returned to participants for comment and/or correction?	No.
Domain 3: analysis and findingsz			
Data analysis			
24.	Number of data coders	How many data coders coded the data?	Three.
25.	Description of the coding tree	Did authors provide a description of the coding tree?	Yes, in the detailed research report.
26.	Derivation of themes	Were themes identified in advance or derived from the data?	Only from the collected data.
27.	Software	What software, if applicable, was used to manage the data?	Maxqda.
28.	Participant checking	Did participants provide feedback on the findings?	No.
Reporting			
29.	Quotations presented	Were participant quotations presented to illustrate the themes / findings? Was each quotation identified? e.*g. participant number*	Yes.
30.	Data and findings consistent	Was there consistency between the data presented and the findings?	Yes.
31.	Clarity of major themes	Were major themes clearly presented in the findings?	Yes.

32.	Clarity of minor themes	Is there a description of diverse cases or discussion of minor themes?	Yes.

Tong A, Sainsbury P, Craig J. Consolidated criteria for reporting qualitative research (COREQ): a 32-item checklist for interviews and focus groups. *Int J Qual Health Care*. 2007 Dec;**19**(6):349–57. doi: 10.1093/intqhc/mzm042.
